# Histomorphometry of penile smooth muscle fiber in severe erectile dysfunction

**DOI:** 10.1590/S1516-31802005000400005

**Published:** 2005-07-07

**Authors:** Joaquim de Almeida Claro, José Aboim, Enrico Andrade, Gustavo Alarcon, Valdemar Ortiz, Francisco Sampaio, Miguel Srougi

**Keywords:** Impotence, Penis, Biopsy, Smooth muscle, Computer-assisted image processing, Impotência, Pênis, Biópsia, Músculo liso, Processamento de imagem assistida por computador

## Abstract

**CONTEXT AND OBJECTIVE:**

Smooth muscle fiber has fundamental importance in erection. Alterations in its function or quantity may be associated with erectile dysfunction. The study objective was to assess the proportion of penile smooth muscle fiber in patients with severe erectile dysfunction.

**DESIGN AND SETTING:**

Clinical study, in the Sexual Dysfunction Group, Universidade Federal de São Paulo (Unifesp), and in the Anatomy Laboratory, Universidade Estadual do Rio de Janeiro (UERJ).

**METHODS:**

Twenty patients with severe erectile dysfunction were selected to form two groups of ten patients: one with normal arterial flow (age range: 44 to 78 years) and the other with altered arterial flow (age range: 38 to 67 years). These groups were compared with a group formed by ten cadavers aged 18 to 25 years that were presumed to have been potent. Quantification of the smooth muscle fibers was done by means of an immunohistochemical study.

**RESULTS:**

The proportion of smooth muscle fiber found was 41.15% for the control group. The patients with erectile dysfunction and normal arterial flow presented 27.24% and those with altered arterial flow presented 25.74%; 19 patients presented at least one chronic disease or risk factor for erectile dysfunction, with prominence for diabetes mellitus, systemic arterial hypertension and smoking.

**CONCLUSION:**

Among patients with severe erectile dysfunction, the arterial flow on its own does not present interference in the proportion of smooth muscle fiber. The diminution of the proportion of smooth muscle fiber may result from chronic diseases and vascular risk factors.

## RESUMO

**CONTEXTO E OBJETIVO:**

A fibra muscular lisa apresenta fundamental importância no mecanismo de ereção e alterações em sua função ou em sua quantidade podem estar associadas à disfunção erétil. O objetivo do estudo é analisar a proporção de fibras musculares lisas em pacientes com disfunção erétil grave.

**TIPO DE ESTUDO E LOCAL:**

Estudo clínico, realizado no Grupo de Disfunção Erétil da Universidade Federal de São Paulo (Unifesp) e no Laboratório de Anatomia e Urologia da Universidade Estadual do Rio de Janeiro (UERJ).

**MÉTODOS:**

20 pacientes com disfunção erétil grave de acordo com o Índice Internacional de Função Erétil e Teste de Ereção Fármaco-Induzida divididos em dois grupos de 10 pacientes, sendo um com fluxo arterial normal (44 a 78 anos, média ≅ 63 anos) e o outro com fluxo arterial alterado (38 a 67 anos, média ≅ 56,2 anos) ao *duplex* ultra-som. Os resultados são comparados com um grupo formado por 10 cadáveres de 18 a 25 anos (média ≅ 21,6 anos) presumidamente potentes. A quantificação das fibras musculares lisas foi realizada a partir de estudo imunoistoquímico com anticorpo anti-actina e análise computadorizada da imagem pelo sistema HSL (matiz, saturação e luminosidade).

**RESULTADOS:**

A proporção de fibras musculares lisas encontradas foi de 41,15% para o grupo controle. Os pacientes com disfunção erétil apresentaram 27,24% de fibras musculares lisas para o grupo com fluxo arterial normal e 25,74% para o grupo com fluxo arterial insuficiente. Com exceção de apenas um paciente (do grupo com fluxo arterial normal), 19 pacientes apresentavam ao menos uma doença crônica ou fator de risco para disfunção erétil, destacando-se *diabetes mellitus*, hipertensão arterial sistêmica e tabagismo, nesta ordem.

**CONCLUSÃO:**

Nos pacientes com disfunção erétil grave, o fluxo arterial isoladamente não apresenta interferência na proporção de fibras musculares lisas, pois os resultados foram semelhantes. Nestes pacientes com disfunção grave, a diminuição das fibras musculares lisas em relação ao grupo controle pode decorrer das doenças crônicas e de seu potencial aterosclerótico.

## INTRODUCTION

Normal penile erection takes place when the smooth muscle of the corpus cavernosum relaxes and the venous return diminishes, thereby holding the blood within the corpus cavernosum. This phenomenon is only possible thanks to the integrity of the tunica that surrounds the corpus cavernosum and, especially, the complete relaxation of the smooth muscle fiber that is responsible for the vein occlusion mechanism.^1,2^ On the other hand, detumescence is caused by contraction of the smooth musculature by means of adrenergic stimulus that also acts on the penile arteries, thereby diminishing their caliber.^3^ In many patients, the etiological basis for erectile dysfunction is found to be related to primary alterations of the smooth muscles of the corpus cavernosum, since its tonus regulates the stages of the erection. The histology and histomorphometry of the corpus cavernosum can be studied by obtaining a specimen of this material. This is generally obtained during surgery for implanting a penile prosthesis, or by means of surgical or percutaneous biopsy.^4-6^

Despite the existence of a considerable number of publications, there are still a lot of discrepancies in the results relating to the proportions of penile smooth muscle fibers in normal individuals and those with erectile dysfunction. This is partly due to the nonuniformity of the techniques utilized and the sample selection methods utilized, which have not always been comparable. The objective of the present study was therefore to determine the real role of smooth muscle fiber in the genesis of severe erectile dysfunction and its correlation with arterial insufficiency.

## METHODS

The sample for the study consisted of 10 cadavers of young men and 20 patients with erectile dysfunction, distributed into the following three groups:

- Group I (control): ten cadavers of young men;
- Group II: ten patients with erectile dysfunction and normal arterial flow as seen on duplex ultrasound;
- Group III: ten patients with erectile dysfunction and arterial insufficiency as seen on duplex ultrasound.

All patients signed an informed consent statement, and the study received the approval of the research ethics committee of Universidade Federal de São Paulo.

The material for group I was formed by specimens of the corpus cavernosum of the penis shaft from cadavers aged between 18 and 25 years. These individuals had suffered violent death caused by car accidents, murder by stabbing or shooting, or falling from a height. The specimens were obtained within 12 hours of death. Cadavers were excluded if these individuals had died of chronic diseases, poisoning or drowning, or if they presented injuries due to physical agents, heat or chemical substances, or if they presented genital lesions. The genitalia were examined to search for the excluding criteria of fibrotic plaque and other alterations, abdominal scars suggesting previous surgery, absence of testicles or testicular volume not in keeping with the individual's age. Cadavers were also excluded if they did not present secondary sexual characteristics compatible with male sex and the individual's age. Therefore, despite the impossibility of being sure that the cadavers were of potent men, the above criteria enable the assumption that they actually were potent. The necropsies were performed at the Instituto Médico Legal (Medical Examiners' Institute) of the State of São Paulo during November and December 1997.

Groups II and III were formed by selecting patients who were attending the outpatient clinic of the erectile dysfunction sector of Hospital São Paulo of the Universidade Federal de São Paulo — Escola Paulista de Medicina (Unifesp-EPM), between February and September 1998. A detailed clinical examination was also performed on each patient, with special attention to underlying diseases and risk factors. The systemic arterial pressure levels were evaluated, and the genitalia were examined in a search for alterations that could exclude them from the study, such as fibrotic plaque or deformities. Blood was collected for performing the following laboratory tests: fasting glycemia, total cholesterol, high-density lipoprotein (HDL), low-density lipoprotein (LDL), triglycerides, total testosterone and prolactin (as seen in Table 4).

The International Index of Erectile Function (IIEF) was applied.^7^ Patients were selected if they had had a complaint of erectile dysfunction for at least one year and if their total score in the erectile dysfunction domain of the IIEF (questions 1, 2, 3, 4, 5 and 15) was a maximum of 6, representing a severe degree of erectile dysfunction.^8^

Next, the patients were submitted to the drug-induced erection test,^9^ using 10 µg of PGE1 injected into the corpus cavernosum. The quality of the erection was graded according to the following criteria:

0. no tumescence

- penile tumescence insufficient for penetration
- penile tumescence sufficient for penetration
- full erection

Patients were included in the study if the drug-induced erection test showed they would be unable to achieve penetration, i.e. they were graded 0 or I.

Finally, the patients were submitted to duplex ultrasound for assessing the systolic peak velocity. From this, two study groups were formed: group II (patients with normal systolic peak velocity) and group III (patients with arterial insufficiency). Results were considered normal when the systolic peak velocity was greater than 30 cm/s, while they were considered to show arterial insufficiency when the systolic peak velocity was less than 25 cm/s.^10-14^ Patients with systolic peak between 25 and 30 cm/s were excluded. In the end, groups II and III were each composed of 10 patients.

Among these patients, specimens from the corpus cavernosum of the penis shaft were obtained under local anesthetic or during surgery for the implantation of a penile prosthesis.

**Figure 1:**
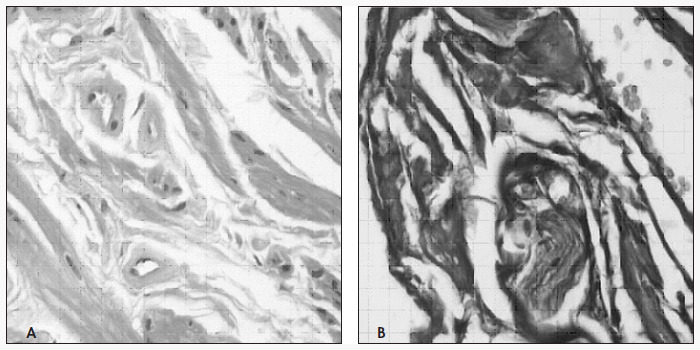
Photomicrographs of the corpus cavernosum, using (A) hematoxylin-eosin staining and (B) Masson trichrome staining, both at 400 x magnification.

Three sections were made per specimen. The first was stained using the hematoxylineosin method for a general analysis of the tissue (Figure 1-A). The second was stained using the Masson trichrome method for identifying the muscle and conjunctive tissue (Figure 1-B).

**Figure 2:**
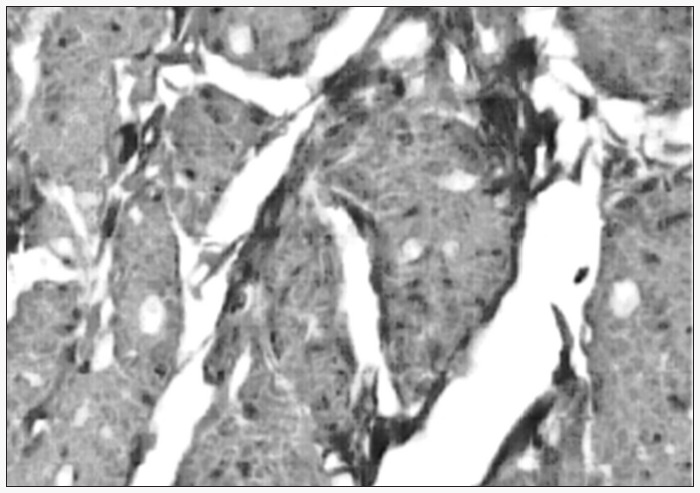
Photomicrograph of the corpus cavernosum with detection of smooth muscle fiber using immunohistochemical analysis (anti-smooth muscle antibodies), 400 x magnification.

The third section was studied by means of the immunohistochemical reaction, using the avidin-biotin complex method. From the avi-din-biotin method, sections were considered to be positive when they presented smooth muscle fiber in tones of chestnut brown, contrasting with a blue background. Differences between the structures within the corpus cavernosum (smooth muscle and collagen tissue) could thus be accentuated (Figure 2).

**Figure 3:**
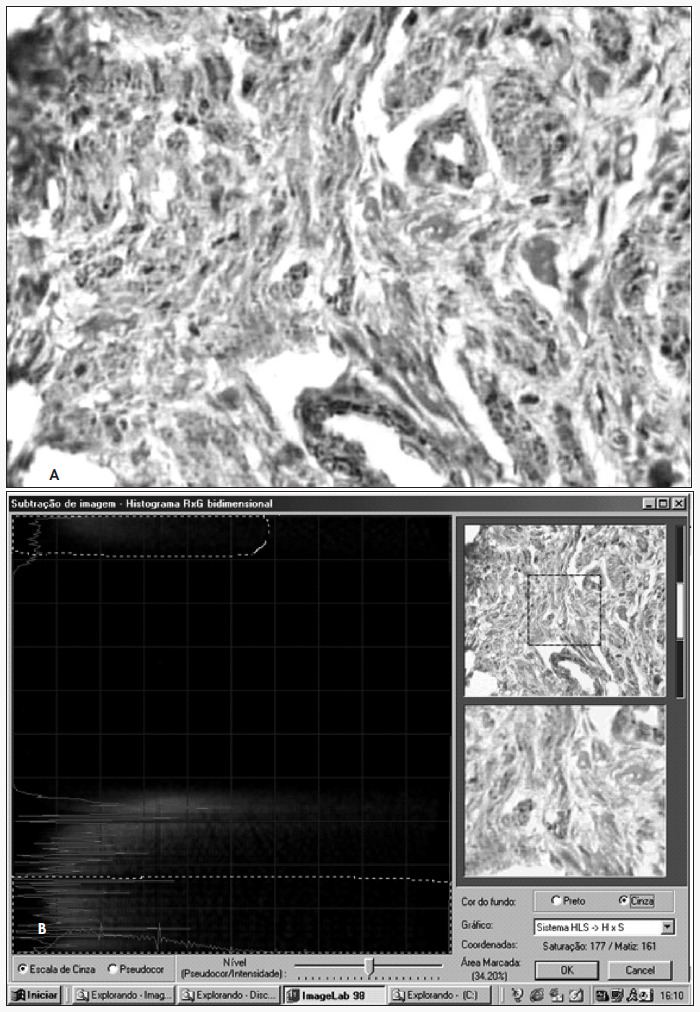
(A) Photomicrograph of the corpus cavernosum subjected to immunohistochemical analysis using anti-smooth muscle antibodies, 400 x magnification. (B) The same histological section during the quantification of smooth muscle fiber: photograph of the image on the computer monitor screen.

To quantify the smooth muscle fiber, each specimen was evaluated in 15 distinct fields. The laminae were analyzed under an optical microscope at a magnification of 400 x. This was done according to the principles of image segmentation and subtraction.^15,16^ The original colors of the images were maintained and transformed into graphs with the colors represented by the hue, saturation and luminosity (HSL). The average value from the 15 measurements was considered to be the proportion of smooth muscle fiber for each penis (Figure 3).

## RESULTS

Out of a total of 146 patients with complaints of erectile dysfunction who were attended, 28 patients (21.91%) met the inclusion criteria. However, eight patients with systolic peak velocity of more than 30 cm/s were lost from the follow-up. Thus, the final composition of groups II and III was 10 patients each.

Table 1 presents the ages and causes of death in group I (the cadavers of young men). In this group the ages ranged from 18 to 25 years (average of 21.6 years). Among the patients (Table 2), the ages ranged from 44 to 78 years (average of 63 years) in group II (normal arterial flow), and from 38 to 67 years (average of 56.2 years) in group III (arterial insufficiency).

**Table 1:** Characteristics of the cadavers (control group) studied

	Age (years)	Cause of death
	18	Internal hemorrhage secondary to bullet wound
	20	Cranioencephalic trauma secondary to bullet wound
	18	Cranioencephalic trauma secondary to car accident
	18	Cranioencephalic trauma secondary to bullet wound
	23	Multiple trauma secondary to car accident
	25	Multiple trauma secondary to car accident
	25	Cranioencephalic trauma secondary to bullet wound
	25	Internal hemorrhage secondary to bullet wound
	20	Cranioencephalic trauma secondary to bullet wound
	24	Internal hemorrhage secondary to bullet wound
Average	21.6	

**Table 2:** Ages (in years), length of time with complaint and grade of erectile dysfunction (ED) among the patients, according to the International Index of Erectile Function (IIEF)

	Group I (control group) Cadavers	Group II Erectile dysfunction Normal arterial flow			Group III Erectile dysfunction Arterial insufficiency		
	Age (years)	Age (years)	No. of years with ED	IIEF grade	Age (years)	No. of years with ED	IIEF grade
	18	78	4	4	67	7	6
	20	68	1	6	55	9	1
	18	62	10	4	61	1	4
	18	55	4	6	58	3	5
	23	66	20	6	38	2	6
	25	54	2	2	57	2	5
	25	63	4	4	62	2	6
	25	44	2	5	56	10	3
	20	68	2	6	62	8	6
	24	72	2	6	46	8	5
Average	21.6	63	5.1	4.9	56.2	5.2	4.7

*Age: Variance analysis by Kruskal-Wallis ranking; p < 0.0001; I < II and III. Length of complaint: Mann-Whitney Test; Group II x Group III; Group II = Group III.*

Table 2 also shows the length of time for which the patients had had their complaint, which ranged from 1 to 20 years in group II (average of 5.1 years) and from 1 to 10 years in group III (average of 5.2 years), and the grade of erectile dysfunction according to the International Index of Erectile Function (IIEF), which ranged from 2 to 6 points in group II (average of 4.9 points) and from 1 to 6 points in group III (average of 4.7 points).

The frequencies of associated diseases and risk factors and the laboratory test results for groups II and III are presented in Tables 3 and 4, respectively.

**Table 3:** Frequency of associated diseases and risk factors in erectile dysfunction groups, via Fisher's exact test (p) or chi-squared test (χ^2^)

Associated diseases (*) and risk factors (**)	Group II Normal arterial flow		Group III Arterial insufficiency		p or χ^2^
	n	%	n	%	
Diabetes mellitus *	6	60.0	7	70.0	p = 0.5000
Arterial hypertension *	6	60.0	5	50.0	p = 0.5000
Coronary insufficiency *	2	20.0	0	–	p = 0.5000
Cerebrovascular event **	2	20.0	0	–	p = 0.2368
Dyslipidemia **	0	–	2	20.0	p = 0.2368
Acute myocardial infarct **	1	10.0	0	–	p = 0.5000
Chronic renal insufficiency *	0	–	1	10.0	p = 0.5000
Arterial surgery **	0	–	1	10.0	p = 0.5000
Smoking **	3	30.0	7	70.0	χ^2^ = 3.20

**Table 4:** Results from the laboratory tests for erectile dysfunction groups (via the Mann-Whitney test)

Laboratory test	Group II Normal arterial flow (average ± SD)	Group III Arterial insufficiency (average ± SD)	^p^
Glycemia (mg/dl)	132.6 ± 34.02	151.9 ± 81.22	0.9705
Total testosterone (ng/dl)	419.2 ± 139.01	486.8 ± 128.48	0.3150
Prolactin (ng/ml)	8.01 ± 2.99	6.65 ± 3.03	0.3930
Total cholesterol (mg/dl)	167.8 ± 33.24	218.8 ± 60.88	0.0288*
HDL cholesterol (mg/dl)	47.1 ± 12.17	49.1 ± 10.21	0.5787
LDL cholesterol (mg/dl)	102.5 ± 27.66	146.4 ± 26.5	0.0015*
Triglycerides (mg/dl)	146.9 ± 67.19	179.0 ± 131.13	0.6842

*SD = standard deviation; HDL = high-density lipoprotein; LDL = low-density lipoprotein;*

* *= significant.*

Table 5 presents the proportion of smooth muscle fiber in the three groups. The range was from 28.92% to 47.63% in group I (average of 41.15%), from 22.16% to 35.51% in group II (average of 27.24%) and from 22.03% to 29.13% in group III (average of 25.74%).

**Table 5:** Proportions of smooth muscle fiber in cadavers and erectile dysfunction patients with normal arterial flow and with arterial insufficiency

	Group I Cadavers	Group II Normal arterial flow	Group III Arterial insufficiency
	44.70	26.92	25.31
	47.19	25.22	28.03
	45.84	28.91	25.37
	43.15	27.31	22.03
	47.63	22.16	29.13
	37.52	26.81	28.66
	38.37	23.99	27.38
	40.05	28.58	24.08
	28.92	35.51	23.91
	38.14	27.02	23.51
Average	41.15	27.24	25.74

*Variance analysis by Kruskal-Wallis ranking; p < 0.0001*

* *I > II and III.*

## DISCUSSION

Muscle tonus regulates the erection, detumescence and flaccidity of the penis. Consequently, the functional and quantitative integrity of the smooth muscle fiber is fundamental for the mechanism of erection.^17^ There may be a decrease in the quantity of smooth muscle fiber with advancing age, with a proportion of as little as 25 to 30%.^18^ of this structure remaining at the age of 75 years. Thus, a study of cadavers without evidence of penile injury found that the quantity of smooth muscle fiber varied as follows: 57% in individuals aged 20 to 40 years, 54% at ages between 40 and 50 years, 51% between 50 and 60 years, 48% between 60 and 70 years and, finally, 44% at ages of between 70 and 80 years.^18^ On the other hand, there are reports giving contrary information, showing that there was no significant difference in the proportion of smooth muscle fiber between individuals without erectile dysfunction and without chronic diseases, comparing two distinct age groups.^19^

In the present study, the average age in group I was 21.6 years, and thus significantly younger than in the two groups of patients with erectile dysfunction: 63 years in group II and 56.2 years in group III, which were comparable with each other. Despite the significant age differences between the groups, we chose this protocol in a attempt to ensure that the cadavers were of potent men.

The parameters of hue and saturation were considered in order to distinguish the smooth muscle tissue from the other structures. Thus, it was ensured that the evaluation would be restricted just to the smooth muscle fibers that had previously been immunologically marked (tones of chestnutbrown color), thereby eliminating the other structures (tones of blue). The proportion was calculated from the area occupied by the smooth muscle fiber in relation to the total area of the field under analysis.^15,16^

Biopsies performed on the corpus caverno- sum also allow the smooth muscle fiber to be quantified. One study found a proportion of smooth muscle fiber of 34% in normal individuals and 30.4% in patients with erectile dysfunction, a difference that was not significant.^20^ On the other hand, the proportion of smooth muscle fiber may reach 40 to 52% in normal men, while being 10 to 36% in individuals with venous-occlusive dysfunction and 13 to 25% in patients with arterial disease.^19^

Values more similar to ours were found in a study that compared the proportion of smooth muscle fiber of the corpus cavernosum in normal individuals and in those with erectile dysfunction.^21^ In this study, the average values were 50% (range: 41 to 61%) and 31% (range: 22 to 46%), respectively. In another study,^22^ the proportions of smooth muscle fiber found in normal men and those with erectile dysfunction were 41.78% for normal individuals, 28.08% for men with erectile dysfunction of venous cause and 23.90% for those with arterial insufficiency. The differences between the three categories presented statistical significance. It was concluded that the ischemic damage would be more severe in patients with arterial dysfunction, thereby causing a more accentuated decrease in smooth muscle tissue.

In the present study, the proportions of smooth muscle fiber found for each group were 41.15% in group I (control), 27.24% in group II (normal arterial flow) and 25.74% for group III (arterial insufficiency). The difference between the group taken to be the control and the patients with erectile dysfunction was statistically significant (p < 0.0001).

The possible mechanisms implicated in the diminution of the quantity of smooth muscle fiber have been studied. In fact, the oxygen pressure within the corpus cavernosum during erection has been found to be lower in patients with severe arteriopathy or diabetic myopathy of the corpus cavernosum who did not respond to prostaglandin injection, despite increased blood inflow.^23^ Furthermore, in such cases, computerized planimetry showed decreased quantities of smooth muscle tissue in the corpus cavernosum.^24^

It has recently been observed^24^ that the oxygen pressure within the corpus cavernosum increases from 25 to 40 mmHg in the flaccid state to 90 to 100 mmHg during erection. Alterations in oxygen levels are thought to be implicated in imbalance between the quantities of smooth muscle fiber and connective tissue. Such balance is fundamental for enabling erection to take place, since it is the connective tissue (collagen) that causes the erection to be maintained. On the other hand, increased collagen associated with diminished smooth muscle fiber leads to an incapability to achieve venous occlusion.^23,24^

The decrease in the proportion of smooth muscle fiber may also be caused by the simultaneous presence of chronic diseases that are associated with erectile dysfunction, such as hypertension, diabetes, coronary insufficiency and chronic renal insufficiency,^25,26^ or by several risk factors like smoking^27^ or hypercholesterol- emia.^28^ Of the 20 patients that we studied, 19 presented at least one morbid condition, and thus only one patient, who was in group II (normal arterial flow), did not present any chronic disease. We therefore did not find any difference between groups II and III, in relation to chronic diseases. Diabetes mellitus was the most frequent finding, affecting six patients in group II and seven in group III. This was followed by systemic arterial hypertension, which affected six patients in group II and five in group III. The other diseases had lesser frequency.

Three patients in group II and seven in group III were smokers. Smoking may be related to greater incidence of arterial insufficiency, although no relation has been found with the proportion of smooth muscle fiber.

The assays of total cholesterol and low- density lipoprotein (LDL) in cholesterol in the present study presented significantly greater values in group III (arterial insufficiency). The total cholesterol levels were 167.8 ± 33.24 mg/dl and 218.8 ± 60.88 mg/dl, respectively, in groups II and III. The LDL cholesterol presented values of 102.5 ± 27.66 mg/dl for group II and 146.4 ± 26.5 mg/dl for group III. In our patients, the increased cholesterol levels were associated with arterial insufficiency. The other laboratory tests performed did not present any significant differences between the groups.

## CONCLUSION

We can conclude that the proportion of smooth muscle fiber found in our control group, with values of 40 to 50%, was within the range of normality. On the other hand, there was a marked decrease in the quantity of smooth muscle fiber among the patients with severe erectile dysfunction.

Thus, it would seem to be correct to suggest that greater care taken with patients who are at risk may diminish the possibility of major irreversible injury to erectile tissue, through the prevention and treatment of conditions that are recognized to have an association with erectile dysfunction.
